# Perineural invasion detection in pancreatic ductal adenocarcinoma using artificial intelligence

**DOI:** 10.1038/s41598-023-40833-y

**Published:** 2023-08-21

**Authors:** Sarah Borsekofsky, Shlomo Tsuriel, Rami R. Hagege, Dov Hershkovitz

**Affiliations:** 1 Institute of Pathology, Tel-Aviv Sourasky Medical Center, 6 Weizmann Street, 6423906 Tel Aviv, Israel; 2 Faculty of Medicine, Tel-Aviv University, Tel Aviv, Israel

**Keywords:** Pancreatic cancer, Image processing, Machine learning

## Abstract

Perineural invasion (PNI) refers to the presence of cancer cells around or within nerves, raising the risk of residual tumor. Linked to worse prognosis in pancreatic ductal adenocarcinoma (PDAC), PNI is also being explored as a therapeutic target. The purpose of this work was to build a PNI detection algorithm to enhance accuracy and efficiency in identifying PNI in PDAC specimens. Training used 260 manually segmented nerve and tumor HD images from 6 scanned PDAC cases; Analytical performance analysis used 168 additional images; clinical analysis used 59 PDAC cases. The algorithm pinpointed key areas of tumor-nerve proximity for pathologist confirmation. Analytical performance reached sensitivity of 88% and 54%, and specificity of 78% and 85% for the detection of nerve and tumor, respectively. Incorporating tumor-nerve distance in clinical evaluation raised PNI detection from 52 to 81% of all cases. Interestingly, pathologist analysis required an average of only 24 s per case. This time-efficient tool accurately identifies PNI in PDAC, even with a small training cohort, by imitating pathologist thought processes.

## Introduction

Pancreatic ductal adenocarcinoma is a malignancy with a bleak prognosis because it is usually diagnosed at a locally advanced or metastatic stage. However, it represents a spectrum of disease severity based on several factors. In 20% of cases, the tumor is resectable based on imaging studies. Overall, these tumors recur 85% of the time with an overall 5-year survival rate of less than 30%^1,2^. The pathologist, however, can stratify the prognosis even further by histological examination of the surgical specimen. One of these prognostic factors is perineural invasion (PNI), which is defined as invasion of the nerve by surrounding tumor cells and/or of tumor penetrating the epineural, perineural, or endoneural space^3,4^. Patients with PNI have a 1–2 year shorter life expectancy^5–7^, most likely because it indicates an increased risk for the presence of occult metastasis. The importance of identifying PNI has also been demonstrated in other cancers such as gastric, breast, and squamous cell carcinoma of the head and neck. It is theorized that the environment of the tissue plane surrounding the nerve provides a conduit of least resistance for the tumor to disseminate, possibly aided by nerve-derived growth factors^8–10^. Due to this microenvironment, PNI has been explored as a potential therapeutic target to prevent the progression of this fatal disease^3,11^. Therefore, the presence or absence of PNI could affect treatment decisions in the future, making its identification even more vital.

Several past studies have demonstrated considerable discordance among pathologists in identifying PNI status. For example, in a study analyzing perineural invasion in squamous cell carcinoma of the vulva, it was shown that 27% of cases inaccurately reported PNI as absent when it was actually present. It was stipulated that this might have been due to the time-consuming nature of analyzing nerves in each patient sample^12^. In another study of PNI in oral squamous cell carcinoma, it was stipulated that the interobserver variability was due to disagreement on the definition of what constituted PNI^13^. Therefore, because of the serious prognostic implications, there is a need for more accurate, reproducible, and time-efficient diagnostic tools.

Artificial intelligence (AI) is an exciting new technology that enables the automation of specific tasks imitating the cognitive learning processes of humans. The more data the AI algorithm is provided, the more reliably the task is performed. Among some of the useful applications of AI are image analysis and being able to identify patterns. In medicine, radiology is an example of a field already utilizing AI algorithms^14–16^. As it has been shown to outperform human radiologists in many instances^17–19^, the increasing incorporation of AI into routine practice in medical fields is anticipated in the future. Digital pathology- the scanning of glass slides for visualization on a computer screen—has recently been adopted by an increasing number of institutions. This move enables digital analysis by artificial intelligence. Indeed, several studies have applied automization in pathology in aspects such as metastases detection in lymph nodes, Ki67 scoring in breast cancer, Gleason grading in prostate cancer, and tumor-infiltrating lymphocyte (TIL) scoring in melanoma^20^. Outperformance of human observers has also been shown, for instance in the detection of lymph node metastasis in women with breast cancer^21^. In addition, AI has been utilized to identify PNI in prostate cancer biopsies^22–24^. Artificial intelligence in pathology is especially useful for laborious tasks that require the identification of very small events in large fields, and identifying PNI in post-Whipple resection specimens with pancreatic cancer is the perfect example of such a task. To date, we are not aware of studies used to identify PNI in pancreatic cancer. In a previous study we applied a novel algorithmic approach termed Hierarchical Contextual Analysis (HCA) to improve detection of ganglion cells^25^. This approach mimics the thought process of pathologists when making a diagnosis. In the present study, we applied the same approach to train the algorithm to identify PNI.

As a result, our paper’s contribution lies in the construction of a highly sensitive artificial intelligence algorithm for detecting PNI in PDAC surgical specimens. Notably, this algorithm also enhances time efficiency and accuracy as compared to a pathologist without our algorithm’s assistance, even with small datasets, highlighting our approach’s practical significance.

## Results

### Training performance analysis

**Figure 1:**
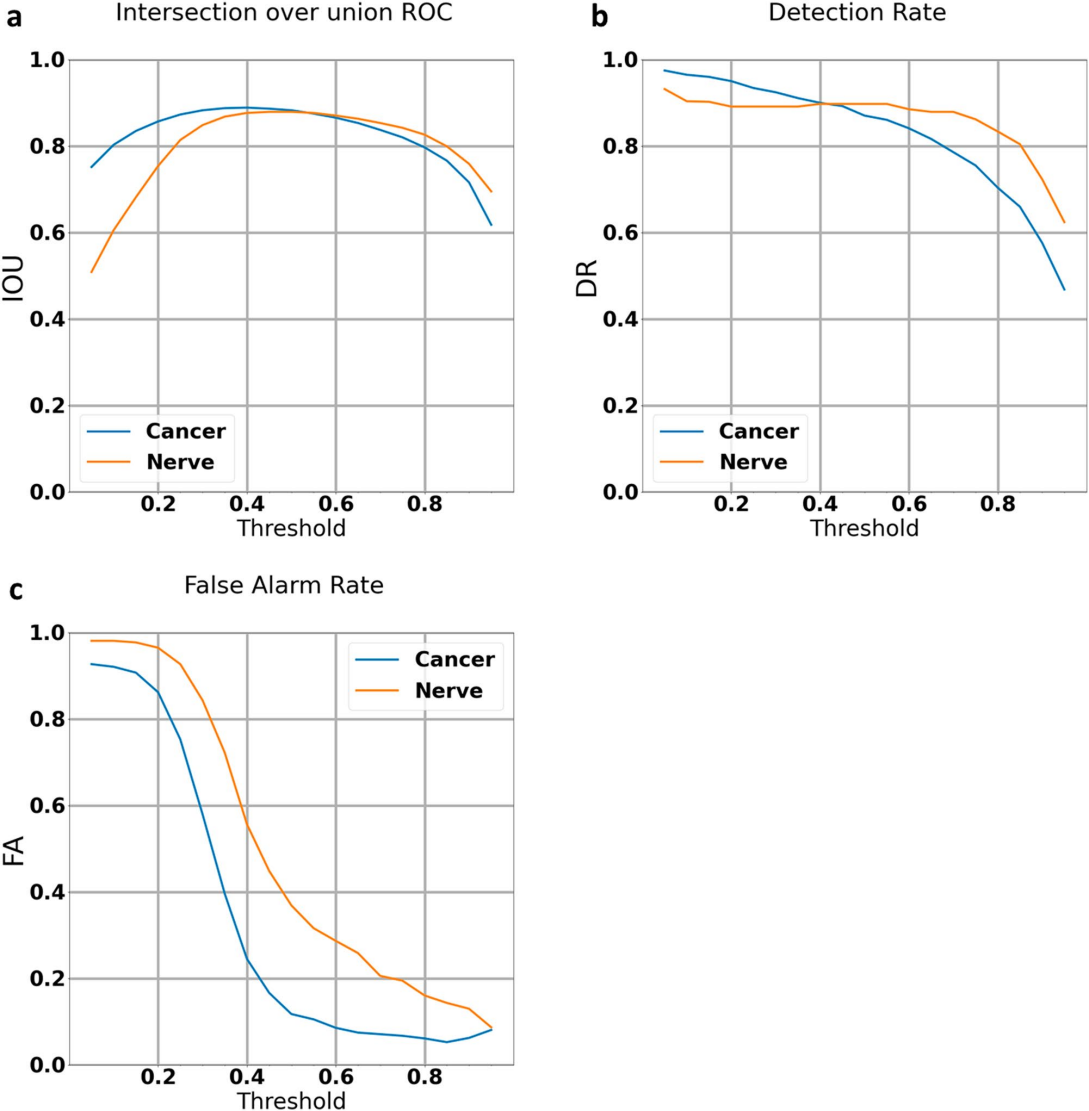
Training set graphs (The algorithm was run on the same 260 labeled images the algorithm was trained on): (**a**) intersection over Union curve: plots the percentage of overlap between the algorithm's and the pathologist's cancer and nerve markings against the confidence threshold. (**b**) Detection rate curve: plots the percentage of cancer and nerve that the algorithm detected against the confidence threshold. (**c**) False Alarm curve: plots the percentage of cancer and nerve that the algorithm falsely identified against the confidence threshold. Results show a relatively high intersection over union and detection rate at mid-range confidence intervals and decreasing false alarms with increased confidence thresholds with higher false alarm rates for nerves than for tumor.

Following training on 260 fully labeled images, the algorithm was initially run on the same 260 images. (See Supplementary material). Algorithm performance showed an Intersection over Union (IOU) of 88 and 88 for nerve and tumor areas at the algorithm confidence threshold of 50% (Fig. 1A). At the same confidence threshold, the detection rates were 90% and 87% for nerve and tumor, respectively (Fig. 1B) and the false alarm rates were 36% and 12% for nerve and tumor, respectively (Fig. 1C).

### Analytical performance analysis

**Figure 2:**
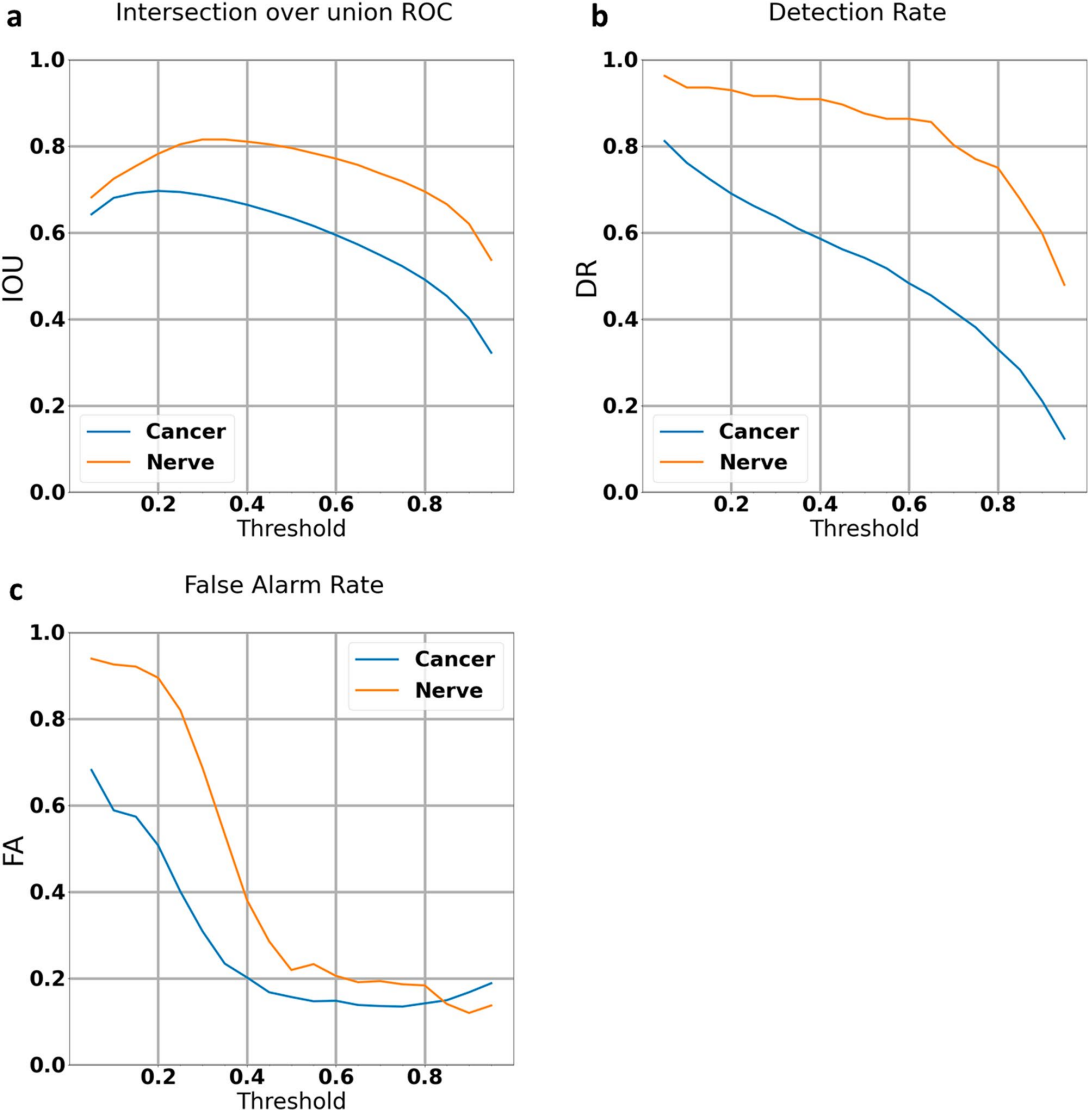
Testing and Verification set graphs: (The algorithm was run on 168 independently labeled images the algorithm was not trained on): (**a**) intersection over Union curve: plots the percentage of overlap between the algorithm’s and the pathologist’s cancer and nerve markings against the confidence threshold. (**b**) Detection rate curve: plots the percentage of cancer and nerve that the algorithm detected against the confidence threshold. (**c**) False Alarm curve: plots the percentage of cancer and nerve that the algorithm falsely identified against the confidence threshold. Steadily decreasing IOU and detection rates are seen with increasing confidence threshold. False alarms are lower for tumor than nerve at confidence intervals of less than 85%.

The algorithm's performance was then evaluated on additional 168 manually labeled images. At the algorithm confidence threshold of 50%, the IOU was 79% for nerve and 63% for tumor (Fig. 2A), the detection rate was 88% for nerve and 54% for tumor (Fig. 2B) and the false alarm rate was 22% for nerve and 15% for tumor (Fig. 2C).

**Figure 3:**
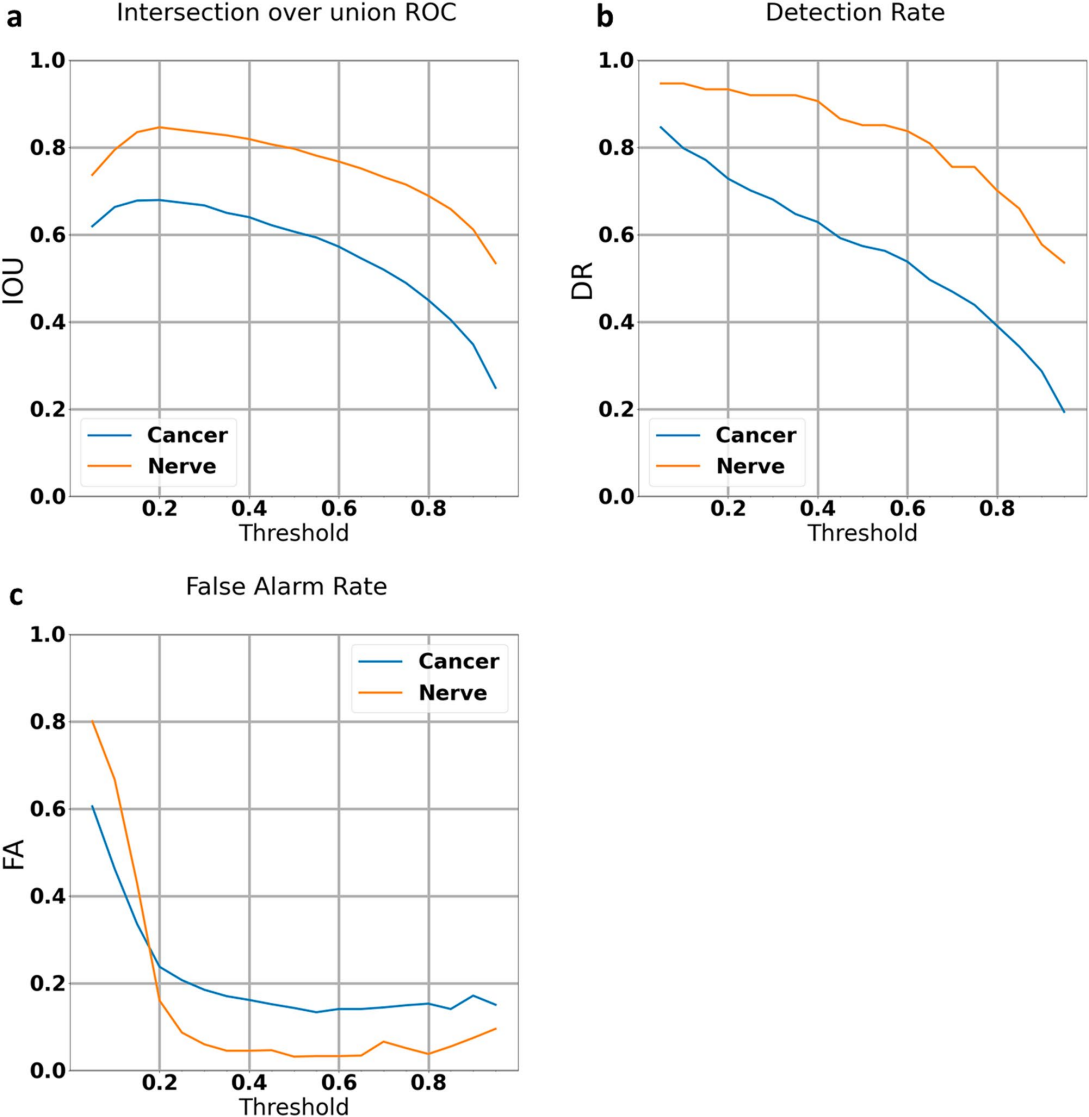
Testing and Verification set graphs after elimination of labels less than 10,000 pixels (The algorithm was run on 168 independently labeled images the algorithm was not trained on): (**a**) intersection over Union curve: plots the percentage of overlap between the algorithm’s and the pathologist’s cancer and nerve markings against the confidence threshold. (**b**) Detection rate curve: plots the percentage of cancer and nerve that the algorithm detected against the confidence threshold. (**c**) False Alarm curve: plots the percentage of cancer and nerve that the algorithm falsely identified against the confidence threshold. Steadily decreasing IOU and detection rates are seen with increasing confidence threshold. Elimination of findings less than 10,000 pixels resulted in a flip where the nerves now have lower false alarm rates than tumor at confidence intervals greater than 18%.

Analysis of the false alarm indications showed that many of those were misclassification of very small foci (less than 10,000 pixels) as tumor. We, therefore, adjusted the algorithm to disregard these findings. Following this adjustment, we got IOU of 79% and 61%, detection rates of 85% and 57%, and false alarm rates of 5 and 15% for nerve and tumor, respectively (Fig. 3).

**Figure 4:**
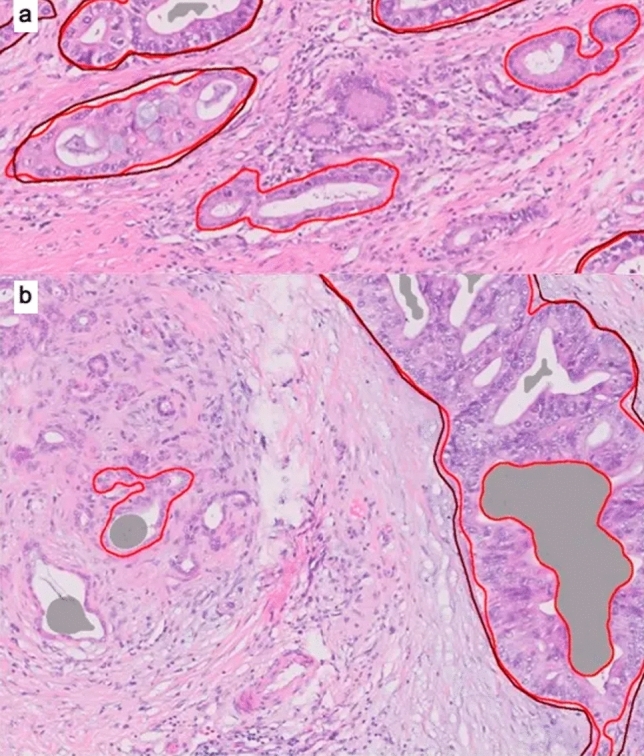
(**a** and **b**) Light red encircled areas without brown encirclement are examples of benign glands falsely designated as cancer by the algorithm.

The results obtained were still not sufficient for clinical application because false alarm rates were too high and detection rates were not high enough. Analysis of the results showed multiple reasons for false alarms. For example, a significant fraction of the false alarm in tumor identification was due to misclassification of benign glands as tumor (Fig. 4). As the clinical application of the algorithm will be based on both identification of PNI elements (nerve and tumor) and their close spatial association, we thought that misclassification of benign glands as tumor would not impair the algorithm’s function. This is because benign glands do not appear in very close proximity to nerves.

### Clinical performance analysis

For the clinical validation, 59 full cases of pancreatic carcinoma were analyzed (1319 slides, 27 ± 9.4 on average per case). In 10 cases, only the slides containing tumor were scanned (61 slides, 6.1 ± 1.8 on average per case). In the pathology reports, 31 (52.5%) cases contained PNI, 26 (44%) were PNI negative. In two cases (3.5%), the pathology report did not contain information regarding PNI—these cases were regarded as PNI negative (total of 28 negative cases, 47.5%). Following analysis by the algorithm, 48 (81.4%) cases contained PNI, and 11(18.6%) were PNI negative. The PNI detection rate was significantly higher after the application of the algorithm (p = 0.00088). Of the 28 cases that were negative based on the pathology report, we were able to detect PNI in 18 cases using the algorithm. Of the 31 positive cases, only one was missed by the algorithm.

**Figure 5:**
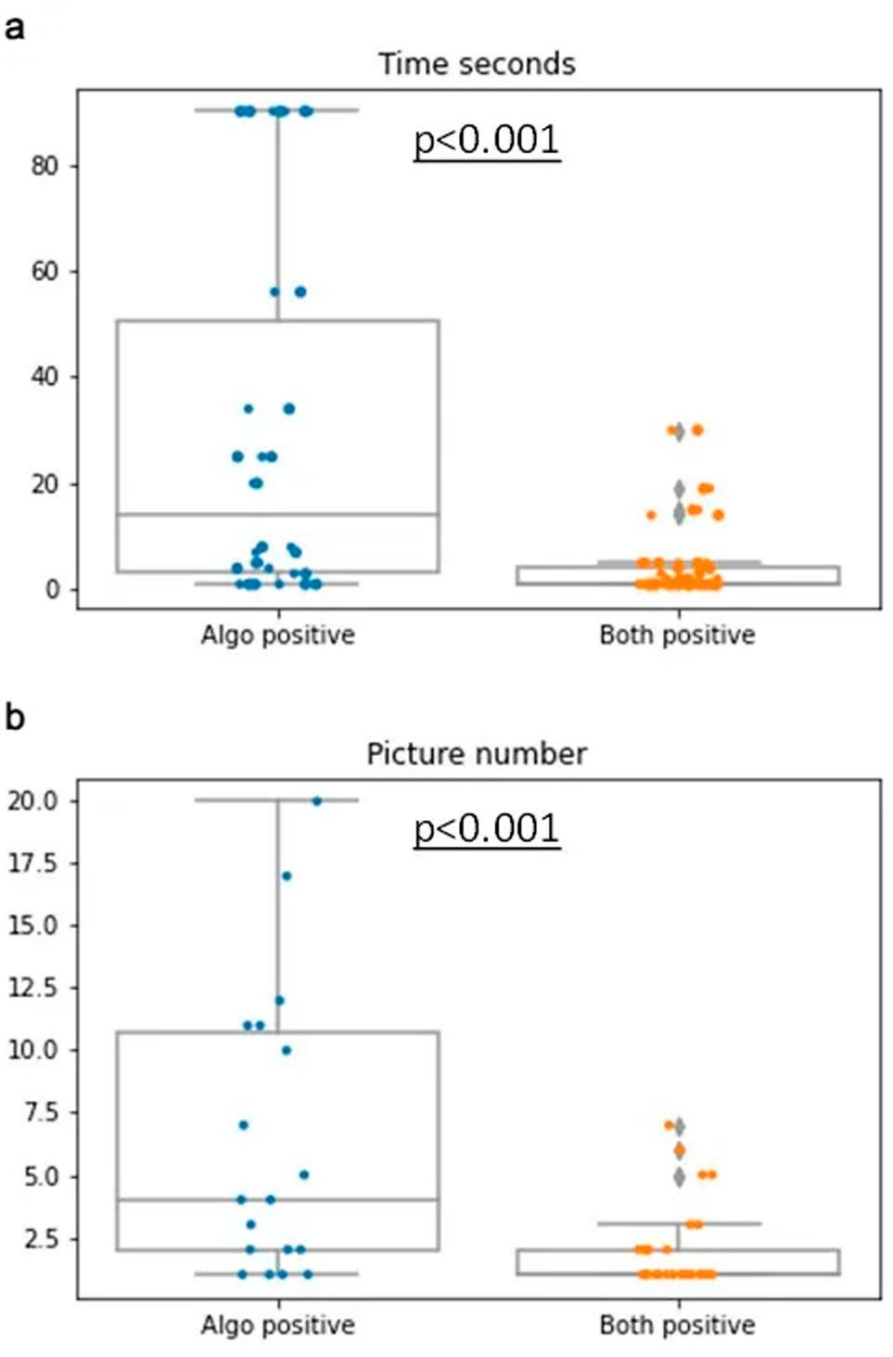
(**a**) Dot plot demonstrating statistically significant more time spent analyzing images where PNI was not originally detected. (**b**) Dot plot demonstrating how the algorithm required more pictures when PNI was not originally detected.

Time for analysis using the images suggested by the algorithm ranged between 1 and 120 s (median 5 s). The number of images containing “clear-cut” PNI per case ranged from 0 to 33 (median 5). Interestingly, in the cases where PNI was identified by the pathologist in the original report, the average time for detecting PNI was 4.2 s with a range of 1–30 s (median 1 s), and the image number where it was detected averaged 0.93 with a range of 1–7 (median 1). In the cases in which the pathologist did not identify PNI originally, the time for detection on the algorithm images averaged 42.4 s with a range of 1–90 s. (median 14 s), and the first positive image number averaged 16.3 with a range of 1–20 (median 4) (Fig. 5). The time and image number were statistically different between both groups (P < 0.001 for both). This indicates that cases that were diagnostically challenging for the pathologist were also more challenging for the algorithm.

## Discussion

Detection of perineural invasion in pancreatic surgical specimens is a laborious and time-consuming process. Even if there is only one instance of PNI around a very small nerve among many slides of tumor, it still changes the PNI status of the patient from negative to positive. Therefore, it is extremely important to scan through every slide very carefully which can add tens of minutes to the diagnostic process. Even then, PNI can still be missed. The difficulty of achieving reliable and consistent results has been demonstrated in several studies showing great variability in the identification of PNI by pathologists^13,26^. This may be due to differences in interpretation of what constitutes PNI and/or differences in time spent examining the slides due to workload/time pressure, etc. It was found that different pathologists interpreted PNI differently depending on what they were taught in residency^13^. These criteria were different because there has been some debate regarding the exact microscopic definition of PNI. Among the different patterns that can be seen are complete and incomplete encirclement as well as tangential contact of the nerve. It is generally accepted that if tumor is seen abutting the nerve outside of the main tumor body, it is considered PNI. If, however, the nerve is inside the main tumor body, several authors have proposed that the tumor needs to encircle at least 33% of the nerve circumference to be considered invasion as opposed to a focal abutment^4,8,27^.

Technology is ripe for assisting pathologists with “needle in haystack” missions, with one common application being mitosis identification^28^. A tool that would standardize the detection of PNI and assist in identifying areas of interest would greatly improve the accuracy and efficiency of the diagnosis/exclusion of PNI. As a result, we felt it was an ideal candidate for automization. Some research has already demonstrated the possibility to do this with prostate cancer^22–24^. For example, Strom et al., showed 87% sensitivity and 97% specificity in the detection of PNI in prostate cancer using digitized and pixel-wise annotated prostate biopsy cores.

In our current study, we developed an algorithm to highlight areas most suspicious for PNI with high sensitivity and sufficient specificity to significantly reduce the workload of the pathologist. Because of the disagreement in the literature regarding the definition of PNI, as described above, it should be noted that our algorithm does not determine the presence or absence of PNI but rather presents to the pathologist the suspicious candidates. Subsequently, the pathologist would determine whether it was true perineural invasion or not rather than the algorithm itself. This eliminated a large number of potential missed events of PNI. However, as a result, this method was not a stand-alone algorithm. A pathologist is still required to make the determination of PNI and the algorithm simply narrowed the fields to areas of interest.

Our study was different from previous studies using artificial intelligence to detect PNI in several ways. First, as far as we are aware, our study is the first attempt at identifying perineural invasion in pancreatic cancer. Second, we used the method of HCA rather than conventional deep learning to eliminate the need for large datasets. Conventional methods of building an image recognition algorithm involve training through using large datasets to identify a desired entity. In our example, this would entail training the algorithm on large numbers of PNI events. Instead, our approach was to train the algorithm to mimic pathologists’ thought processes by training the system to detect nerve and tumor fibers as separate groups and then asking the algorithm to identify places where these groups were in close proximity to each other. This method allowed us to get around the requirement for extensive amounts of data generally needed in conventional deep learning techniques to achieve medically satisfactory results. As a result, specific algorithms can be built and tailored within hospitals without the need to resort to multi-institutional studies that require vast resources and numbers of specimens. This makes AI more accessible and could incorporate the use of AI into general practice more quickly. This approach has already been successfully applied to detect ganglion cells as a part of the diagnosis of Hirschprung disease^25^.

The algorithm constructed in this study significantly shortened the time needed for a pathologist to determine PNI status many-fold from tens of minutes using conventional means, to less than a minute. In a study by Egevad et al.^26^ analyzing inter-observer variability in PNI detection in prostate core biopsies, they found that the median time for analyzing a core biopsy for PNI was 81 s. One can extrapolate the extra time it would take to analyze multiple slides from a surgical specimen.

Not only was the algorithm able to shorten the detection time, but it was also successful at detecting PNI more accurately and reliably, highlighting areas of PNI in 97% of the cases in which PNI was previously diagnosed. Additionally, the algorithm identified PNI in 18 cases previously reported as PNI-negative. In the cases where PNI had originally been missed by conventional methods, the average time and number of images that needed to be analyzed to detect PNI were greater than in cases with an unmissed diagnosis of PNI (P < 0.0001). We believe this suggests that the algorithm improved the detection of PNI in cases that were more difficult to find and/or when PNI was a rarer event. This further lends credence to the idea that this novel method is a potentially useful tool for pathologists to identify PNI in pancreatic cancer.

This study had a few limitations which merit mention. While already being very effective at detecting PNI, training with larger datasets with a greater variety of cases including from other institutions, as well as using different scanning platforms would improve the accuracy and reproducibility of the algorithm even further^29,30^. On the flip side, the fact that the algorithm performed so well at identifying PNI from such a small dataset points to the strengths of this algorithm development method.

In addition, while the algorithm performed with high sensitivity and specificity for identifying nerves, it was less specific in identifying tumor cells. Following analysis, it was found that one of the reasons for false positives was because many events classified as tumor were in fact benign glands. This type of error is of minimal importance due to the fact that even a benign-appearing gland close to a nerve is, by definition, tumor. Therefore, a benign gland would not be identified in the event of PNI. This type of input from a pathologist (i.e. a gland adjacent to a nerve or blood vessel is tumor by definition) could significantly help an AI algorithm more accurately differentiate between benign and malignant glands as well.

To improve the accuracy and reduce the number of false positives, there was a pixel cutoff for the minimum size of an event to be included in the algorithm detection. This could result in the algorithm missing particularly small instances of perineural/intraneural invasion.

Future potential projects would involve efforts to train the algorithm with larger datasets, improved pixel threshold cutoffs, improved identification with prioritization of the most suspicious areas, and correlation with clinical outcomes using different variables (e.g. size of the nerve, frequency of PNI). Perineural invasion detection could also be broadened for use in other types of cancer. The training used to identify tumor could also be expanded to train an algorithm to detect invasion of tumor in lymph nodes, another very important consideration of tumor staging.

## Conclusion

We were able to construct an artificial intelligence algorithm to assist pathologists with the detection of perineural invasion in pancreatic adenocarcinoma surgical specimens with high sensitivity while significantly increasing time efficiency and accuracy compared to pathologist analysis alone. This was achieved even with small datasets which highlights the utility of our algorithm-building method.

## Methods

All methods were performed in accordance with the relevant guidelines and regulations.

### Ethics statement

All data used in this study was derived from digital pathology slides identified only by a sample number and no other identifying details. The study was approved and informed consent was waived by the local ethics committee at Tel-Aviv Sourasky medical center (ethics approval #TLV-16-660).

### Clinical samples

The material used in this research was derived from formalin-fixed paraffin-embedded tissue. Hematoxylin and eosin-stained slides were scanned using the Philips UFS scanner (Koninklijke Philips, Amsterdam, The Netherlands), at X40 magnification. The proprietary ISYNTAX format was converted to TIFF format using the Philips IntelliSite pathology Solution program, version 3.2, and annotations were manually made, by use of a locally developed annotation tool. The pathological ground truth was based on hospital records. All cases were previously reviewed by senior pathologists, most with expertise in pancreatic pathology.

### Research design

**Figure 6:**
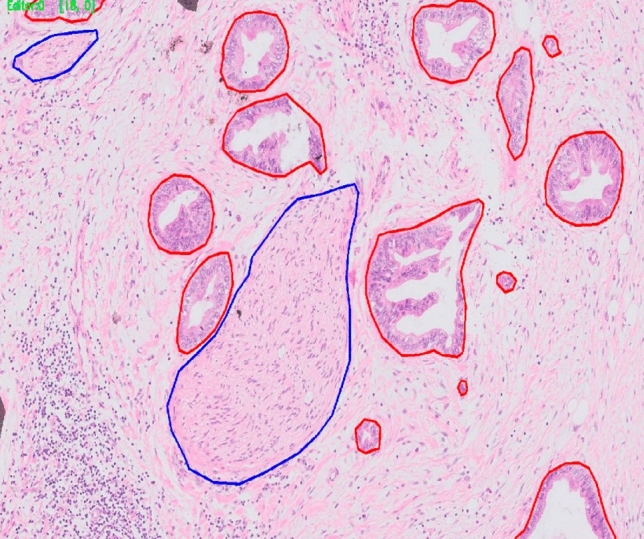
Example of labeling done by a pathologist .Red, tumor. Blue, nerve.

The research included a training step, an analytical performance analysis step, and a clinical performance analysis step. Data from the images included direct annotation of tumor and nerves (Fig. 6) as well as the remainder of the image designated as negative-labeled background.

### Algorithmic approach and training

After scanning the slides of 6 cases of previously diagnosed invasive pancreatic adenocarcinoma, 260 fields were selected for the algorithm's training. 204 of the fields were selected randomly, and 56 fields of relevant areas with higher numbers of nerve and tumor events were selected by a pathologist.

**Figure 7:**
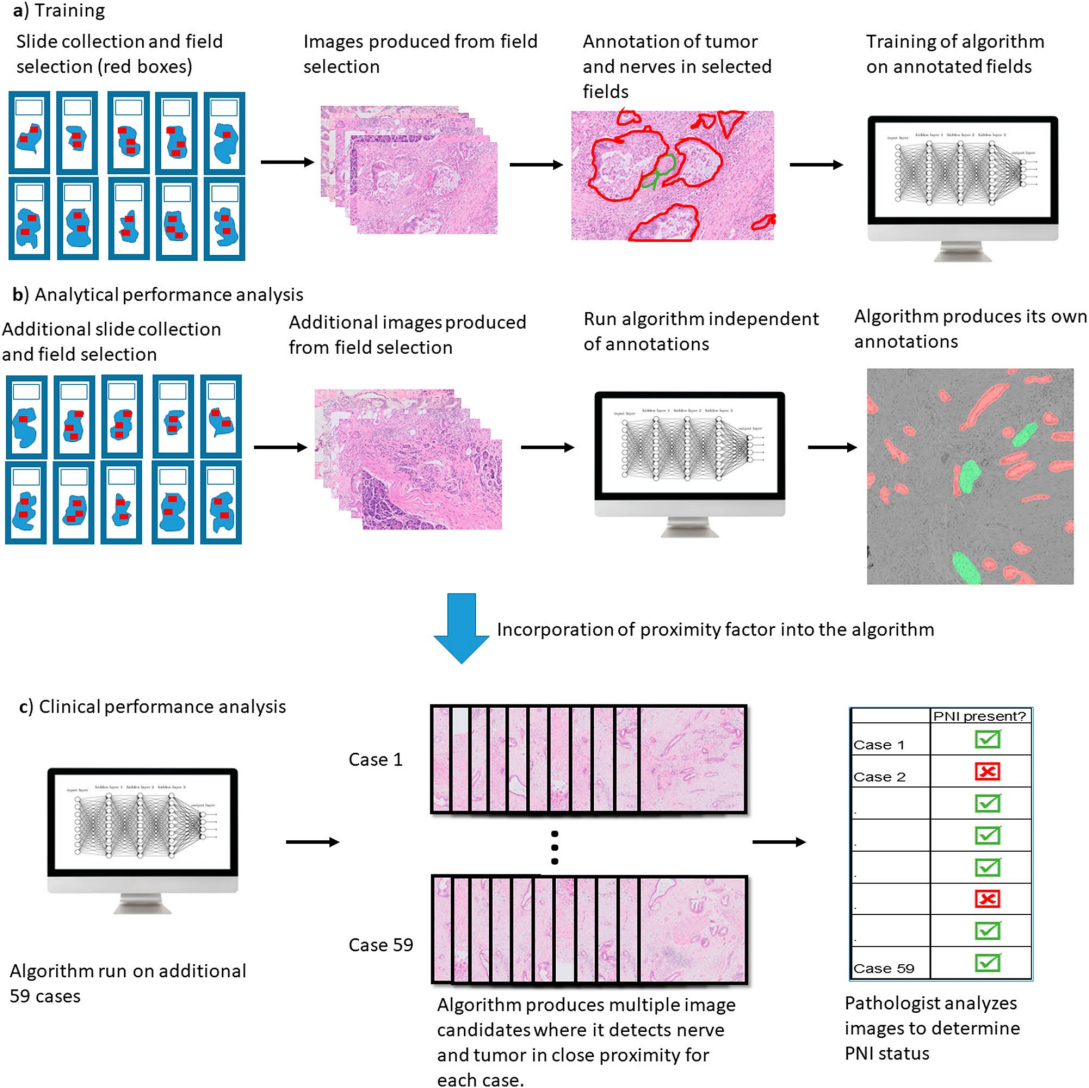
Methods schematic: (**a**) For the algorithm training phase, slides were collected and fields were selected for manual annotation of tumor and nerves. The algorithm was then trained on these annotated fields. (**b**) For the algorithm analytical performance analysis fields, additional fields were selected, however, the algorithm was run independently of these annotations to produce annotations of its own. (**c**) The algorithm was then run on an additional 59 cases to produce fields of areas where it detected nerve and tumor in close proximity. The images were then analyzed to evaluate the presence or absence of PNI.

To develop the model we first used the pathologist’s insights and raw data without any annotation, namely fully unsupervised learning, in order to create the algorithm framework and degrees of freedom that the algorithm has. The framework integrated convolutional neural networks (CNN) and decision processes inspired by expert knowledge. U-Net CNN structure was used as a first approximation of the desired result. Subsequently, all slides were marked by a single pathologist who manually segmented nerves and tumor structures (Fig. 6). Unmarked fields were regarded as negative for tumor and nerve. We then tailored the system to the given annotations of the 260 pictures by training the deep neural networks. Data augmentation was designed to improve robustness of the algorithm, partially via the use of generative adversarial networks (GANs). Afterward, the algorithm was run on these same images and we determined the intersection over union (degree of overlap between the algorithm and the pathologist markings), the detection rate, and the rate of false alarms (Fig. 7).

### Analytical performance analysis

To test the algorithm, we generated 168 additional fully labeled fields that were not used for training. The algorithm ran on these images and the degree of intersection over union, detection rate, and rate of false alarm were determined.

### Clinical performance analysis

Fifty-nine cases of pancreatic adenocarcinoma with a total of 1319 slides were used for the validation phase. Ten of the cases had only the slides with tumor scanned and the remaining cases had the entire case scanned. The algorithm presented the 20 areas most suspicious for PNI for the 10 initial cases and for the remaining 39 cases, the 40 areas most suspicious for PNI. This was done by having the algorithm first identify tumor and nerves above a set confidence level providing a score of 0 if it was below the set confidence level and 1 if it was above the set confidence level. Afterward, the 20 or 40 images with the smallest distances between nerve and tumor were presented. A pathologist analyzed these images and determined whether PNI was present or absent in each case.

### Supplementary Information

Supplementary Figure 1.

Supplementary Legend.

## Data Availability

The datasets used and/or analyzed during the current study are available from the corresponding author on reasonable request, pending institutional review board approval.
