# Supplementary figures and images for: Perineural invasion detection in pancreatic ductal adenocarcinoma using artificial intelligence
Source: Sci Rep. 2023 Aug 21;13:13628. doi: 10.1038/s41598-023-40833-y (PMC10442355; doi:10.1038/s41598-023-40833-y)

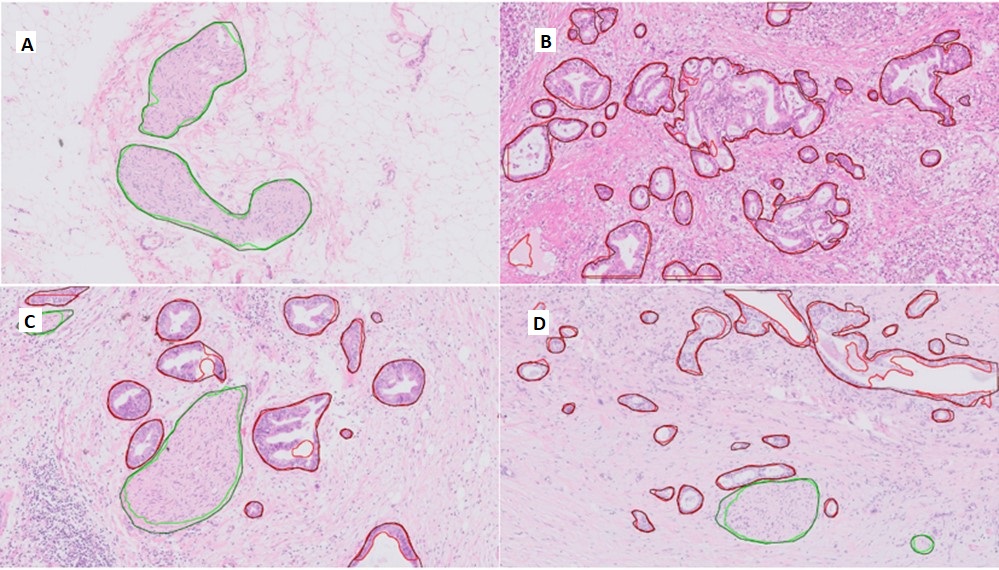

Supplement: Supplementary file 1 — Supplementary Figure 1. [file 41598_2023_40833_MOESM1_ESM.jpg]
