# Supplementary material for: Perineural invasion detection in pancreatic ductal adenocarcinoma using artificial intelligence
Source: Sci Rep. 2023 Aug 21;13:13628. doi: 10.1038/s41598-023-40833-y (PMC10442355; doi:10.1038/s41598-023-40833-y)
Supplement: Supplementary file 2 — Supplementary Legend. [file 41598_2023_40833_MOESM2_ESM.docx]

Supplemental Figure 1: Images generated after the algorithm performed an analysis on the 260 previously labeled slides that were used for training. (A) Field with only nerve (B) Field with only tumor (C+D) Fields with both nerve and tumor. Dark green: Nerves labeled by pathologist. Brown: Tumor labeled by pathologist. Light green: Nerves labeled by algorithm. Dark green: Tumor labeled by algorithm. These figures show that subjectively, there was a high concordance in the areas detected by the algorithm and the pathologist's labels, which is expected due to the algorithm being trained on the same labeled set. It also shows that the training set was successfully building the algorithm.
